# Consensus on covert awareness: a Delphi study

**DOI:** 10.1093/braincomms/fcaf462

**Published:** 2025-11-21

**Authors:** Caroline Schnakers, Berno Overbeek, Niko Fullmer, Liliana Teixeira, Matteo Zandalasini, Kseniia Yatsko, Ann-Marie Morrissey, Nathan Zasler, Anna Estraneo

**Affiliations:** Research Institute,Casa Colina Hospital and Centers for Healthcare, Pomona, CA 91767, USA; Department of Primary and Community Care, Radboud University Medical Center, Research Institute for Medical Innovation, Nijmegen, 6525 GA, The Netherlands; Department of Rehabilitation, Azora, Terborg, 7061 AP, The Netherlands; Research Institute,Casa Colina Hospital and Centers for Healthcare, Pomona, CA 91767, USA; Center for Translational Health and Medical Biotechnology Research (TBIO)/Health Research Network (RISE-Health), Polytechnic Institute of Porto, School of Health, 4200-465 Porto, Portugal; Dipartimento di Medicina Riabilitativa, Azienda USL di Piacenza, Unità Spinale, Neuroriabilitazione e Medicina Riabilitativa Intensiva, 29121 Piacenza, Italy; Faculty of Fundamental Medicine, M.V. Lomonosov Moscow State University, 119991 Moscow, Russia; School of Allied Health, Health Research Institute, Ageing Research Centre, University of Limerick, Limerick, V94T9PX, Ireland; Department of Physical Medicine and Rehabilitation, Virginia Commonwealth University, Richmond, VA 23284, USA; Department of Rehabilitation, Don Gnocchi Foundation IRCCS, 50143 Florence, Italy

**Keywords:** consciousness, vegetative state, unresponsive wakefulness syndrome, minimally conscious state, covert awareness

## Abstract

Identifying wilful brain activity in patients with disorders of consciousness is critical, as some patients fail to exhibit behavioural signs of consciousness at the bedside but respond to active tasks via neuroimaging or electrophysiological measures. Standardized terminology for this subgroup is absent while it is essential for advancing research and clinical care. The objective of this study was to determine the level of consensus among a large group of international experts on terminology and definitions for this clinical entity, as described by terms such as covert awareness, cognitive motor dissociation, functional locked-in syndrome, and non-behavioural minimally conscious state. A Delphi study was conducted using REDCap to evaluate expert agreement on terminology and definitions. The study was conducted among international experts, primarily from Europe/UK, the USA and other regions. Ninety-six experts participated. Among these, 75 (78%) completed both rounds. Participants were predominantly clinical scientists (71%) working in rehabilitation settings (63%). A Delphi method was followed. Consensus on terminology and related definitions was defined as a median score of 5, an interquartile range ≤1 and ≥75% agreement (scores of 4 or 5). Within two rounds, consensus was achieved for over two-thirds of the statements. The term ‘Covert Awareness’ and its associated definition were identified as the preferred terminology by an international expert panel. We recommend the use of ‘Covert Awareness’ since our large group of international experts consistently agreed on such preferred term for this subgroup of patients with disorders of consciousness. This consensus (>75% agreement) establishes a foundation both for future research and clinical standardization. The findings have implications for improving diagnostic accuracy and advancing understanding of covert awareness, although further study is needed to refine and apply the agreed-upon definition in clinical practice.

## Teaser

Schnakers *et al*. conducted an international Delphi study with a large panel of experts and reached consensus on the term ‘Covert Awareness’ for patients with disorders of consciousness showing wilful brain activity without behavioural signs. This consensus represents a major step towards research standardization and clinical management of such patients.

## Graphical Abstract

**Graphical Abstract:**
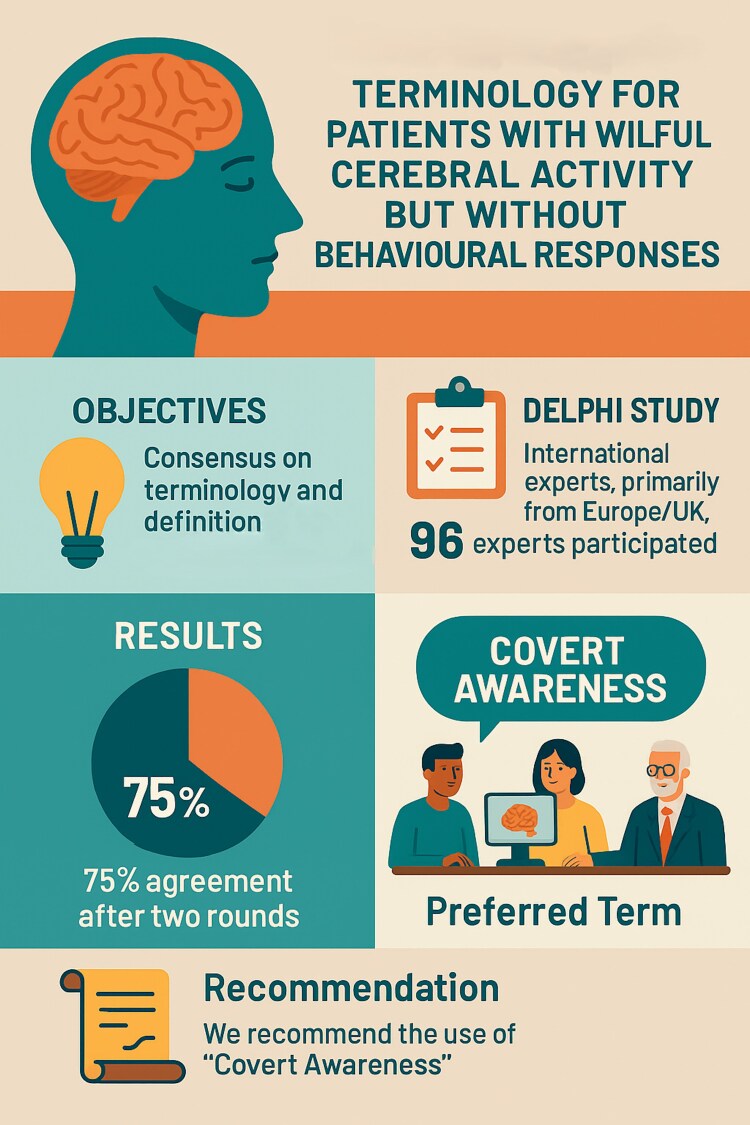

## Introduction

Recognizing behavioural indicators of consciousness in individuals with severe acquired brain injury is often difficult, and the absence of sensitive assessment tools can lead to misdiagnosis in nearly 40% of cases.^1^ Even when structured bedside evaluations are used, some patients with disorders of consciousness (DoC) remain unable to display wilful behaviour because of profound motor impairments. Indeed, over the last two decades, a subgroup of patients who, while behaviourally unresponsive, demonstrate purposeful brain activity when tested with active neuroimaging (functional MRI, fMRI) or electrophysiological paradigms has repeatedly been described in the literature. The first evidence of this phenomenon was reported in 2006, describing the case of a young woman diagnosed with vegetative state/unresponsive wakefulness syndrome (VS/UWS; i.e. wakefulness without behavioural evidence of awareness).^1^ When asked to imagine playing tennis, her brain activity on fMRI mirrored that of healthy controls.^2^ A subsequent study using the same paradigm in a larger cohort (*n* = 54) found that two patients clinically diagnosed as VS/UWS and three diagnosed as minimally conscious state (MCS; inconsistent yet reproducible signs of awareness)^1^ successfully performed the task. Moreover, one patient used motor and spatial imagery to answer autobiographical yes/no questions.^3^ Since then, numerous reports have confirmed the existence of this phenomenon. Meta-analyses estimate that 14–17% of patients classified as VS/UWS show evidence of covert awareness (CA),^4,5^ and a recent multicentre investigation reported rates as high as 25%.^6^ These findings prompted both the American and European Academy of Neurology to recommend the use of additional diagnostic techniques such as neuroimaging and electrophysiology when assessing patients with DoC.^7,8^

Although the clinical reality of this condition is acknowledged, there is no agreement on the terminology used to describe it. A recent systematic review identified 25 different labels, many of which are applied inconsistently—even within single publications. Among the most frequently used are CA, cognitive motor dissociation (CMD), functional locked-in syndrome (fLIS), and non-behavioural MCS (MCS*).^9^ Each of these terms have been defined in different ways in the existing literature calling for a consensus on the best clinical label for the condition. Indeed, using a clear taxonomy, along with precise definitions, has several key benefits. It supports rigorous research among scientists, facilitates effective communication and diagnostic consistency among healthcare professionals and upholds trust and professionalism when interacting with families and the broader public.^10^ A precise taxonomy would therefore be helpful for both scientists and clinicians.

In this context, the present Delphi study aimed to examine the level of agreement among a large group of international experts on these terms and their related definitions across the available literature. The overarching goal of our study was to inform future discussions on taxonomy for this condition.

## Materials and methods

Based on an extensive literature review,^9^ a Delphi method was used to investigate the level of consensus on names and related definitions for CA, CMD, fLIS and MCS*. A Delphi study is a multi-staged survey, which aims to achieve consensus on an important issue.^7,11,12^ The result is an expert opinion about a subject where previously no such opinion existed. In the development of clinical guidelines, the Delphi method is used as a consensus building process to capture expert opinions and experiences.^7^ This study adheres to the DELPHISTAR recommendations for Delphi studies.^11^

### Survey development

Since international consensus was aimed for, a core group of five members from the Special Interest Group on DoC of the International Brain Injury Association (IBIA DoC SIG) was formed. The members of this group are all experts in their respective country (USA, the Netherlands, Ireland, Russia, Portugal and Italy) and participated in previous projects on the same topic.^5,9^ Additionally, one member (B.O.) had previous experiences with using the Delphi method.^12^ The core group developed the survey but did not participate in the study. The study protocol survey data were collected and managed using Research Electronic Data Capture (REDCap) hosted at Casa Colina Hospital and Centers for Healthcare. REDCap is a secure, web-based software platform designed to support data capture for research studies.

The survey included, across rounds, 11–16 statements related to definitions and 2–3 questions related to name preferences. For each statement, the level of agreement was indicated on a five-point Likert scale as used in Delphi studies:^11,12^ strongly disagree (1), moderately disagree (2), neutral (3), moderately agree (4) and strongly agree (5). A comment section was also included for each definition and was used for additions and/or revisions of statements in the second round. The statements used for each definition were based on a previous systematic review^9^ that was accessible to each participant using a direct link to the article embedded in the survey. Before starting the Delphi process, a pilot was done among core members to test the survey and estimate completion time. The survey duration was kept <30 min to decrease risks of incomplete surveys or drop-outs (Supplementary Table 1).

### Expert criteria

After the core group approved the final version of the survey, an international panel of experts were identified and selected using one or more/one of the following criteria: (i) at least 5 years of research and/or clinical experience in the assessment and treatment of patients with a DoC, in the acute, post-acute or chronic phases and (ii) published at least five peer-reviewed articles on DoCs over the last 5 years. The core group agreed that a large sample would be needed not only to increase our chances to recruit experts fitting our criteria but also to optimize the reliability (less influence of outliers, more resilience to drop-outs), as well as the generalizability of our results. Three hundred experts were contacted, based on the rate of participation (45%) and loss-to-follow-up (25%) in a previous Delphi study involving experts in DoC.^12^ To reach this high number of potential experts, participants were recruited by contacting IBIA DoC SIG members and by snowball sampling.

### Data collection

Based on the Delphi method,^11,12^ the core group agreed on a two to three round of data collection, since this approach provides the best balance between obtaining a consensus and maintaining a high response rate. The core group also agreed that a third round would not be performed if consensus was reached for more than two-thirds of the statements on Round 2 and that a maximum of three rounds would be done even if consensus for agreement could not be reached for more than two-thirds of these statements.^11,12^ Each identified expert received an email invitation including an individualized link, gave digital informed consent before starting the first-round and were then given access to the survey. Responses were anonymized in REDCap; all responders being related to a number upon starting the survey. Demographic data were collected in Round 1 to document the participants’ profile and to ensure a fit with our criteria for expertise (i.e. country of residence, profession, experience working with DoCs in years, number of relevant publications, work setting, use of assessment scales and use of additional diagnostic techniques). Participants had the possibility to ‘save and return later’ after starting the survey to prevent fatigue among responders (i.e. potential consensus bias). After submitting feedback, each participant received an email with a pdf summarizing their responses, which they were asked to review just before answering next round of the survey. The feedback provided was aggregated across all expert groups. Each round was open for 3 weeks (with an automatic reminder after 7 days) and had 1-week (Round 2) to 2 weeks (Round 1) interval in-between rounds to allow time for analysis. The analyses were done by the core group. The Delphi study took place between the beginning of April until the end of May 2024.

### Statistical analysis

Based on the Delphi method,^11,12^ measures of central tendency and dispersion were used. Consensus was also calculated by combining median values, interquartile ranges (IQR), and percentage of agreement/disagreement. The median values for per cent agreement were weighted in terms of the number of respondents. As done in most Delphi studies, consensus for agreement was defined as a median score of 5, an IQR ≤ 1 and ≥75% scoring 4 or 5 while consensus for disagreement was defined as a median of 1, an IQR ≤ 1 and ≥75% scoring 1 or 2.^11,12^ If consensus for agreement (or disagreement) was reached for a statement, this statement was not presented again in the next round. The core group was advised by experts (B.O.) from Radboud University Medical Center regarding statistics.

The protocol was reviewed and approved by the Institutional Review Board (IRB) committee of Casa Colina Hospital and Centers for Healthcare, Pomona, CA, USA.

## Results

### Expert panel

**Figure 1:**
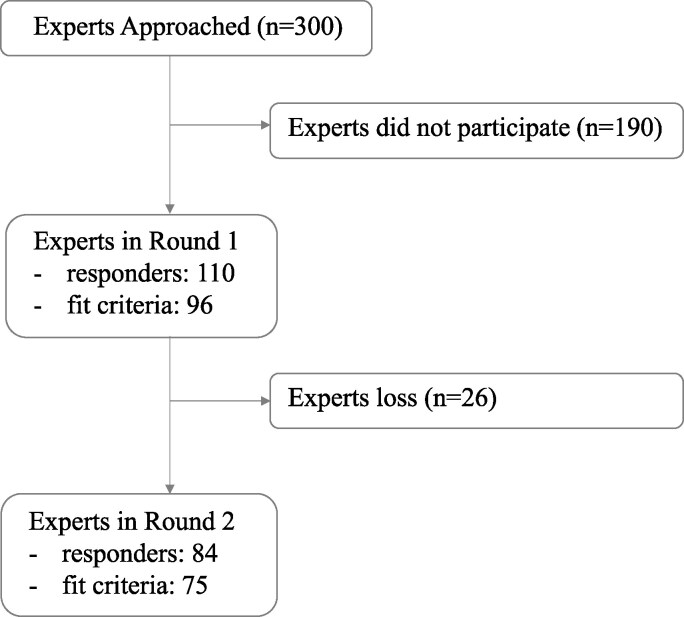
Flow chart of participation of experts.

Ninety-six experts agreed to participate and fit our criteria for expertise. Seventy-five experts (78%) completed all rounds (Fig. 1). Most experts were clinical scientists (i.e. medical experts who study new techniques to help prevent, diagnose and treat illness) (71%). Participants worked as medical doctors (51%; specialized in neurology, intensive care medicine, neurosurgery and physical medicine and rehabilitation), psychologists (13%), physical therapists (5%), occupational therapists (5%), speech therapists (3%), registered nurses (2%) as well as (neuro) scientists (20%). Most experts (64%) worked in a rehabilitation setting, 45% in an acute setting, 23% in a long-term care setting and 8% in a research setting. Forty-nine per cent were located in Europe and UK (EU/UK), 32% in the USA and 19% from other parts of the world (Others; i.e. Russia, Canada, Australia, Israel, Brazil, Malaysia and China). When diagnosing DoCs, behavioural assessments were the most frequently used (90%) by these experts, followed by electrophysiology [67% EEG and 42% event-related potentials (ERP)], neuroimaging (34% fMRI, 27% PET, 7% single-photon emission computed tomography—SPECT). Other techniques such as TMS-EEG (21%—combining transcranial magnetic stimulation and EEG) or brain computer interface (BCI; 13%) were also used. Active paradigms were less frequently used (28%) than resting-state (48%) or passive paradigms (37%) (Table 1).

**Table 1:** Participants characteristics

Variables	*n*	%
Country of residence		
USA	31	32
EU/UK	47	49
Others	18	19
Russia	6	6
Canada	5	5
Australia	2	2
Israel	2	2
Brazil	1	1
Malaysia	1	1
China	1	1
Profession		
Medical doctor	49	51
Psychologist	13	14
Physical therapist	5	5
Occupational therapist	5	5
Speech therapist	3	3
Nurses	2	2
Clinical scientist	68	71
Neuroscientist	19	20
Setting		
Acute	43	45
Rehabilitation	61	64
Long-term care	22	23
Research	8	8
Population		
Paediatric (<18 years)	22	23
Adult (18–64 years)	91	95
Elderly (65 years and above)	62	65
Technique access		
Behavioural	86	90
EEG	64	67
ERP	40	42
fMRI	33	34
PET	26	27
SPECT	7	7
Transcranial magnetic stimulation combined with EEG–TMS-EEG	21	22
BCI	13	14
Technique paradigm		
Passive listening	36	38
Active tasks	27	28
Rest	46	48

### Consensus development

This Delphi study included two rounds without deviation to protocol. Indeed, a third round was not necessary since consensus was reached for more than two-thirds of the statements on Round 2 (cf. Delphi methodology described above).^11,12^

#### Round 1

Consensus was only reached for 5 out of the 16 (31%) definition statements (2/5 for CA, 1/3 for CMD, 2/5 for fLIS and 0/3 for MCS*) (Table 2). Also, no consensus was reached on questions related to name preferences (Table 3). For the definition statements, all statements that did not reach consensus were rephrased for Round 2 based on the experts’ feedback. The responses in the free-text comments were analysed independently by two reviewers (C.S., B.O.). For name preferences, one question related to the choice of a covert-related name (i.e. CA, covert consciousness and covert cognition) was also included in Round 2 based on this feedback.

**Table 2:** Statements with agreement rates in Rounds 1 and 2

	Mdn	IQR	*n*	% agree	*Consensus*
CA					
Round 1					
Non-communicative patients	4	2	90	72%	N
Including those diagnosed as vegetative, minimally conscious, or locked in	4	3	89	52%	N
Who may be able to use their residual cognitive capabilities	5	1	88	82%	**Y**
To communicate their thoughts to those around them	3.5	3	88	50%	N
By modulating their own neural activity	5	1	88	77%	**Y**
Round 2					
Patients who are in a disorder of consciousness and who do not show functional communication verbally or by gestures	5	1	74	84%	**Y**
Including those behaviourally diagnosed as in a VS/UWS	5	1	74	80%	**Y**
Who may be able to use their residual cognitive capabilities	*	*	*	*	**Y**
To follow command and/or communicate	5	1	74	80%	**Y**
By modulating their own neural activity	*	*	*	*	**Y**
CMD					
Round 1					
Severely brain-injured patients	5	1	81	84%	**Y**
Including patients in VS (or UWS), in minimally conscious state minus (i.e. without reproducible response to command), or complete locked-in syndrome	5	3	81	63%	N
Who show a dissociation of a retained but unrecognized (covert) cognitive capacity	5	2	81	72%	N
Round 2					
Severely brain-injured patients	*	*	*	*	**Y**
Including patients behaviourally diagnosed as VS (or UWS) or minimally conscious state minus (i.e. without reproducible response to command)	5	1	71	86%	**Y**
Who show a dissociation of a retained but unrecognized (covert) consciousness using active neuroimaging and/or electrophysiological paradigms	4	3	71	63%	N
Non-behavioural MCS (MCS*)					
Round 1					
Patients who are diagnosed as being in a VS (or UWS) by bedside testing	5	2	81	65%	N
But then diagnosed MCS with neuroimaging techniques	4	3	81	57%	N
Using passive or active paradigms	4	2	81	59%	N
Round 2					
Patients who are initially diagnosed as being in a VS or UWS by behavioural assessment	5	1	71	82%	**Y**
But whose brain activity is similar to MCS with neuroimaging and/or electrophysiology	4	3	71	65%	N
Using active tasks	4	2	71	69%	N
fLIS					
Round 1					
A dissociation between motor dysfunctions and preserved higher cognitive functions	5	1	83	77%	**Y**
As shown by functional imaging techniques	4	2	83	72%	N
and a consistent and reliable communication	4	2	83	72%	N
Using non-speech and non-gestural communication	5	1	82	82%	**Y**
Through direct brain signalling	5	2	82	73%	N
Round 2					
A dissociation between motor dysfunctions and preserved higher cognitive functions	*	*	*	*	**Y**
As shown by functional neuroimaging and/or electrophysiology	5	1	73	82%	**Y**
And a reliable communication	5	2	73	74%	N
Using non-speech and non-gestural communication	*	*	*	*	**Y**
Through brain modulation	5	2	73	68%	N

Mdn, median; IQR, interquartile range; *n*, sample size; Consensus (no/N, yes/Y in bold for readability); *, statement not included in Round 2 (consensus reached).

**Table 3:** Results related to the question: ‘To what extent do you agree with the following name(s)?’

	Mdn	IQR	*n*	% agree	Consensus
Round 1					
Before taking the survey					
CA	4	1	95	78%	N
CMD	4	2	95	69%	N
Non-behavioural MCS (MCS*)	3	2	95	35%	N
fLIS	2	2	95	22%	N
After taking the survey					
CA	4	1	81	77%	N
CMD	4	2	81	67%	N
Non-behavioural MCS (MCS*)	3	2	81	44%	N
fLIS	2	3	81	26%	N
Round 2					
Before taking the survey					
CA	5	1	75	95%	**Y**
CMD	4	2	75	65%	N
Non-behavioural MCS (MCS*)	2	3	75	27%	N
fLIS	1	1	75	8%	**Y**
After taking the survey					
CA	5	1	71	87%	**Y**
CMD	4	2	71	73%	N
Non-behavioural MCS (MCS*)	2	3	71	30%	N
fLIS	2	2	69	12%	N
Covert-related names					
CA	5	1	71	83%	**Y**
Covert consciousness	4	2	71	66%	N
Covert cognition	3	2	71	34%	N

CA, covert awareness; CMD, cognitive motor dissociation; MCS*, non-behavioural MCS; fLIS, functional locked-in syndrome; Mdn, median; IQR, interquartile range; *n*, sample size; Consensus (no/N, yes/Y in bold for readability).

#### Round 2

**Figure 2:**
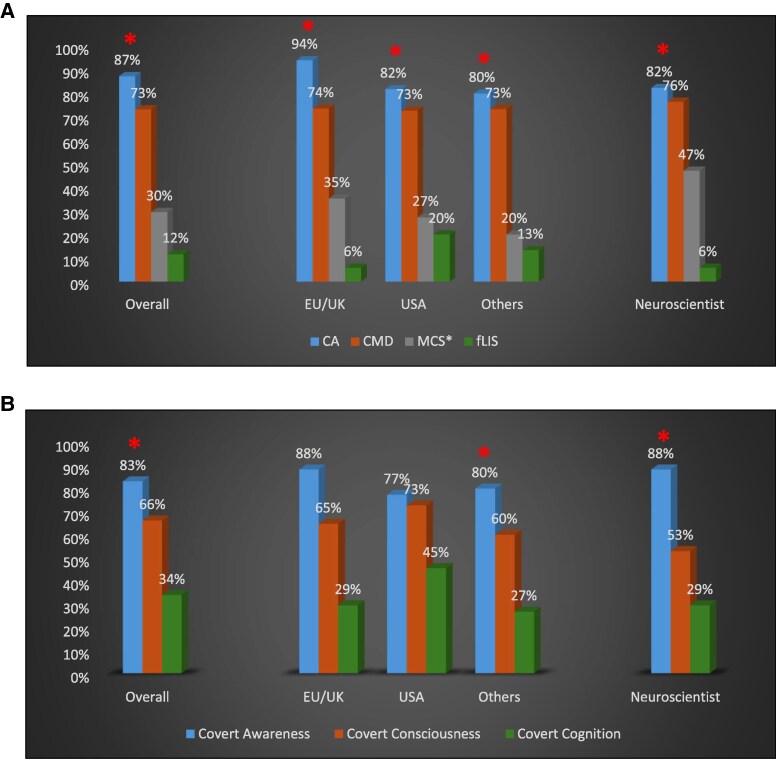
**Consensus.** Consensus (*) overall, according to geographic location (EU/UK, USA, and Others) and background (neuroscientist) for CA, CMD, MCS* and fLIS (**A**) as well as for covert-related names (**B**). CA, covert awareness; CMD, cognitive motor dissociation; fLIS, functional locked-in syndrome; MCS*, non-behavioural MCS.

Consensus for agreement was reached for 11 out of the 16 (69%) statements (5/5 for CA, 2/3 for CMD, 3/5 for fLIS and 1/3 for MCS*). A full consensus was reached on a definition for CA (Table 2). Moreover, a consensus only for CA was reached when experts were asked which name they would agree upon (87%), and which covert-related names they would prefer (83%). A consensus for disagreement was reached on fLIS in Round 2 for questions related to name preferences (84%) (Table 3). When considering background and only selecting neuroscientist experts, same results were found; i.e. a consensus for all statements of the definition for CA only and a consensus on CA as the name preferred for this condition (over CMD, MCS* and fLIS as well as over covert consciousness or covert cognition) (Fig. 2; Supplementary Tables 2 and 3). The consensus was also compared among geographic locations (USA versus EU/UK versus Others, Supplementary Tables 2 and 3). A consensus on CA as the name preferred for this condition was consistently found (over CMD, MCS* and fLIS) across all locations. Compared with other covert-related names (such as covert consciousness or covert cognition), the highest agreement was observed for the term CA (>75%) but a consensus was not reached among US and EU/UK responders. Regarding CA definition, a consensus was reached for all statements among responders of EU/UK versus Others, while two statements did not reach consensus among US responders (Fig. 2; Supplementary Tables 2 and 3).

## Discussion

This international study examined the level of agreement on the nomenclature and related definitions for a clinical entity that was identified almost 20 years ago^2^ and has received various names through the years without any consensus among experts.^9^

Even though this study did not aim to reach a consensus on such a challenging topic, our results show that the term CA (i.e. ‘covert awareness’) quickly reached a consistent consensus (>75% agreement) among the large group of international experts involved in this study (96 in Round 1 and 75 in Round 2). A consensus was also reached on its definition; i.e. ‘patients who are in a disorder of consciousness and who do not show functional communication verbally or by gestures, including those behaviourally diagnosed as in a VS/UWS, but who may be able to use their residual cognitive capabilities to follow command and/or communicate by modulating their own neural activity’.

Most of our experts were clinical scientists (71%). Since this background was over-represented, secondary analyses were performed focusing on a subgroup of experts with a neuroscience background. These analyses showed consistent results with a consensus on CA definition and on CA as the name preferred for this condition (over CMD, MCS*, fLIS, covert consciousness or covert cognition). Geographic locations were also compared and showed slightly different results. A consensus across locations was reached on CA as the preferred name over CMD, MCS* and fLIS but, despite CA receiving the highest agreement (>75%), a consensus was not reached on CA as the preferred name over covert consciousness or covert cognition among US and EU/UK responders. Finally, a consensus for all statements of CA definition was observed among responders of EU/UK and Others but not among US responders with two of the statements not reaching consensus.

Despite slight differences, our results show a consistent result across backgrounds and locations: a consensus on CA as the preferred name over CMD, MCS* and fLIS. The term CA was originally used by Owen and his collaborators (located in the UK) in one of the first papers on the topic and is one of the most highly referenced in the literature.^9^ However, such a quick consensus was rather surprising since ‘Cognitive Motor Dissociation’ or CMD is frequently used in the DoC literature. This name was first mentioned by Schiff *et al*. (located in USA) in 2015 in response to a publication by Fernández-Espejo *et al*.,^13^ who showed structural disconnection between thalamus and primary motor cortex in one ‘covertly aware’ patient. Schiff^14^ wrote an editorial on these results and suggested the term CMD to account for ‘… the sharp dissociation of a retained but unrecognized (covert) cognitive capacity in some severely brain-injured patients with non-purposeful or absent behavioral responses’. This term has been used in recent high-profile publications^6,10^ suggesting it was adopted by the field. To the contrary, the current Delphi study demonstrates otherwise as no consensus was reached on the name CMD and its definition. Based on the experts’ feedback, most of the debate was about: (i) the vagueness of the definition, which could apply to completely unrelated pathologies such as LIS^1^ or amyotrophic lateral sclerosis and (ii) more importantly, the fact that such a name implies that cognition is intact (which is currently unknown) and which could lead to misunderstanding among researchers, healthcare professionals, or when interacting with families and the broader public. Other terms such as MCS* and fLIS failed to reach consensus. Most experts criticized MCS* for the inclusion of a passive paradigm to detect CA which do not assess wilful brain activity and might lead to false positives. The term ‘functional locked-in’ or fLIS was the only term that reached consensus for disagreement as most experts found it confusing and disliked the direct comparison with (classical/incomplete) LIS that has well-defined neuropathology and a different clinical profile.^1^

Our results also showed that, when diagnosing DoCs, techniques such as EEG (67%) are more frequently used by clinical experts as compared with functional neuroimaging (fMRI, 34%). Active paradigms that are usually involved in detecting wilful brain activity are used in a minority of cases (28%) when compared with resting state (48%), which is consistent with prior findings.^4^ This reflects the reality of clinical versus research settings. Indeed, even though it is possible to use cheaper bedside techniques such as EEG in most clinical settings, it is challenging to use active paradigms as it requires a level of expertise often unavailable in non-academic settings. These challenges have been previously highlighted in the literature and call for the development of centres of expertise that clinicians could refer to, when implementing paradigms and analysing their data.^10^ This is particularly needed since a recent multicentric study including 353 patients found that 25% of patients without an observable response to commands (and 20% of patients diagnosed in coma and VS/UWS) demonstrated CA.^6^ Moreover, not only diagnostic techniques but also treatment strategies should be developed and/or implemented (e.g. using BCI or neuromodulation techniques) for a condition identified almost 20 years ago. However, for research to go further, we not only need a better understanding of the biomarkers related to this condition^10,15^ but also, and foremost, a clear taxonomy to allow a better communication between experts.

This study nevertheless has some limitations. Our results were stable across background, when comparing clinical scientists to neuroscientists. However, it would be interesting to confirm our findings in a bigger sample of neuroscientists to ensure reliability since the sample included in this study was relatively small (*n* = 17). Also, part of our responders was from non-native English-speaking countries which could have affected our findings. Our results were comparable for the preferred name (CA) across locations (e.g. USA, EU/UK and Others) but the level of agreement for definition statements related to CA differed across locations. Further work might therefore be needed on the definition of CA itself in the future. Third, one could criticize the fact that definitions related to other covert-related terms were not included in this study. Indeed, based on an extensive literature review,^9^ CA was chosen since it was the most frequent term used in the literature, among all covert-related terms. Moreover, when other covert-related names (i.e. covert consciousness and covert cognition) were included in Round 2, a consensus only on CA was still obtained. In any case, future discussions should address in which context CA should be applied and whether the use of other terms should be avoided or applied to distinct, separate situations. For example, one could imagine that while CA would apply to a VS/UWS who respond to command using active neuroimaging tasks, CMD might apply to patients who reliably communicate using such tasks and who should therefore be considered out of DoC (cf. Aspen criteria for the emergence from MCS).^1^ Finally, an interesting aspect that has not been tackled in this study is the opinion of caregivers of patients with DoC. Ensuring that a chosen taxonomy is accepted by experts and non-experts is the ultimate step since such a choice can impact not only caregivers but also public understanding of this condition.

In conclusion, based on our results, we recommend the use of CA since our large group of international experts consistently agreed on such term for this subgroup of patients with DoC. This consensus, based on high agreement (>75%), establishes a foundation both for future research and clinical standardization. The findings have implications for improving diagnostic and prognostic accuracy as well as advancing understanding of CA in DoC, although further study is needed to refine and apply the agreed-upon definition in clinical practice.

## Supplementary Material

fcaf462_Supplementary_Data

## Data Availability

De-identified participant data will be accessible upon request to researchers whose proposed data use has received approval from an ethical committee for a specified purpose. Access will be granted by the corresponding author (cschnakers@casacolina.org) following the completion of a signed data access agreement.
