# Supplementary material for: Consensus on covert awareness: a Delphi study
Source: Brain Commun. 2025 Nov 21;7(6):fcaf462. doi: 10.1093/braincomms/fcaf462 (PMC12671493; doi:10.1093/braincomms/fcaf462)
Supplement: fcaf462_Supplementary_Data [file fcaf462_supplementary_data.pdf]

## Supplementary Material

**Supplementary Table 1.** Survey questions for Rounds 1 and 2

| **Round 1** | |
|---|---|
| **Country of Residence** | |
| **Profession** | Medical Doctor |
| | Psychologist |
| | Physical Therapist |
| | Occupational Therapist |
| | Speech Therapist |
| | Neuroscientist |
| | Professor |
| | Other |
| | Are you a clinical scientist (both working as a clinician and doing research)? |
| **Inclusion criteria** | When considering the last ten years, how many years of experience do you have with the assessment and/or treatment of patients with disorders of consciousness (DoC)? |
| | When considering the last ten years, how many years of experience do you have in research on patients with DoC? |
| | How many articles (available on Pubmed) did you publish on DoC in the last 5 years? |
| **Setting** | Acute |
| | Rehabilitation (inpatient and/or outpatient) |
| | Long-term care (nursing home, skilled nurse facility - SNF, long term acute care - LTAC) |
| | Other |
| **Population** | Pediatric (< 18yo) |
| | Adult (18-64yo) |
| | Elderly (65yo and above) |
| **Technique access** | Behavioral |
| | Electroencephalogram–EEG |
| | Event-related potentials–ERP |
| | Functional magnetic resonance imaging – fMRI |

Positron emission tomography–PET
Single-photon emission computed tomography–SPECT
Transcranial magnetic stimulation combined with EEG–TMS-EEG
Brain computer interface–BCI
N/A
Other

**Technique paradigm**
Passive listening
Active tasks
Rest
N/A
Other

**Before answering the survey, to what extent do you agree with the following name(s)**

Covert awareness (CA)
Functional locked-in (fLIS)
Non-behavioral MCS (MCS*)
Cognitive motor dissociation (CMD)
*Comments:*

**To what extent do you agree with parts of the definition below?**

***CA definition***
non-communicative patients
including those diagnosed as vegetative, minimally conscious, or locked in
who may be able to use their residual cognitive capabilities
to communicate their thoughts to those around them
by modulating their own neural activity
*General comments on the name Covert Awareness (CA)*

***fLIS definition***
a dissociation between motor dysfunctions and preserved higher cognitive functions
as shown by functional imaging techniques
and a consistent and reliable communication
using non-speech and non-gestural communication comments
through direct brain signaling
*General comments on the name Functional Locked-In Syndrome (Functional LIS)*

| | |
|---|---|
| ***MCS* definition*** | patients who are diagnosed as being in a vegetative state (VS; or unresponsive wakefulness syndrome - UWS) by bedside testing |
| | but then diagnosed MCS with neuroimaging techniques comments |
| | using passive or active paradigms comments |
| | *General comments on the name Non-behavioral Minimally Conscious State (MCS*)* |
| ***CMD definition*** | severely brain-injured patients |
| | including patients in vegetative state (VS; or unresponsive wakefulness syndrome - UWS), in minimally conscious state minus (or MCS-; i.e., without reproducible response to command), or complete Locked In Syndrome (LIS) |
| | who show a dissociation of a retained but unrecognized (covert) cognitive capacity |
| | *General comments on the name Cognitive Motor Dissociation (CMD)* |
| **After answering the survey, to what extent do you agree with the following name(s)** | |
| Covert awareness (CA) | |
| Functional locked-in (fLIS) | |
| Non-behavioral MCS (MCS*) | |
| Cognitive motor dissociation (CMD) | |
| *Comments* | |
| **Round 2** | |
| **Before answering the survey, to what extent do you agree with the following name(s)** | |
| Covert awareness (CA) | |
| Functional locked-in (fLIS) | |
| Non-behavioral MCS (MCS*) | |
| Cognitive motor dissociation (CMD) | |
| *Comments* | |
| **To what extent do you agree with parts of the new definition below?** | |
| ***CA definition*** | patients who are in a disorder of consciousness and who do not show functional communication verbally or by gestures |
| | including those behaviorally diagnosed as in a Vegetative State/Unresponsive Wakefulness syndrome |
| | to follow command and/or communicate |
| | *General comments on the name Covert Awareness (CA)* |

| | |
|---|---|
| ***fLIS definition*** | as shown by functional neuroimaging and/or electrophysiology |
| | and a reliable communication |
| | through brain modulation |
| | *General comments on the name Functional Locked-In Syndrome (Functional LIS)* |
| ***MCS* definition*** | patients who are initally diagnosed as being in a vegetative state (VS; or unresponsive wakefulness syndrome - UWS) by behavioral assessment |
| | but whose brain activity is similar to Minimally Conscious State (MCS) with neuroimaging and/or electrophysiology |
| | using active tasks |
| | *General comments on the name Non-behavioral Minimally Conscious State (MCS*)* |
| ***CMD definition*** | including patients behaviorally diagnosed as vegetative state (VS; or unresponsive wakefulness syndrome - UWS) or minimally conscious state minus (or MCS-; i.e., without reproducible response to command) |
| | who show a dissociation of a retained but unrecognized (covert) consciousness using active neuroimaging and/or electrophysiological paradigms |
| | *General comments on the name Cognitive Motor Dissociation (CMD)* |
| **After answering the survey, to what extent do you agree with the following name(s)** | |
| Covert awareness (CA) | |
| Functional locked-in (fLIS) | |
| Non-behavioral MCS (MCS*) | |
| Cognitive motor dissociation (CMD) | |
| *Comments* | |
| **To what extent do you agree with the following "covert" related name(s)?** | |
| Covert awareness | |
| Covert consciousness | |
| Covert cognition | |
| *Comments* | |

**Supplementary Table 2.** Definition statements with agreement rates for CA, CMD, MCS*, fLIS, according to geographic location (EU/UK, USA, Others) and background (neuroscientist)

| **EU/UK** | ***Mdn*** | ***IQR*** | ***n*** | ***% agree*** | ***Consensus*** |
|---|---|---|---|---|---|
| patients who are in a disorder of consciousness and who do not show functional communication verbally or by gestures | 5 | 1 | 36 | 89% | **y** |
| including those behaviorally diagnosed as in a Vegetative State/Unresponsive Wakefulness syndrome | 5 | 1 | 36 | 81% | **y** |
| to follow command and/or communicate | 5 | 0 | 36 | 81% | **y** |

| **USA** | | | | | |
|---|---|---|---|---|---|
| patients who are in a disorder of consciousness and who do not show functional communication verbally or by gestures | 5 | 2 | 23 | 74% | n |
| including those behaviorally diagnosed as in a Vegetative State/Unresponsive Wakefulness syndrome | 5 | 1 | 23 | 78% | **y** |
| to follow command and/or communicate | 5 | 2 | 23 | 74% | n |

| **Others** | | | | | |
|---|---|---|---|---|---|
| patients who are in a disorder of consciousness and who do not show functional communication verbally or by gestures | 5 | 1 | 15 | 87% | **y** |
| including those behaviorally diagnosed as in a Vegetative State/Unresponsive Wakefulness syndrome | 5 | 1 | 15 | 80% | **y** |
| to follow command and/or communicate | 5 | 1 | 15 | 87% | **y** |

| **Neuroscientist** | | | | | |
|---|---|---|---|---|---|
| patients who are in a disorder of consciousness and who do not show functional communication verbally or by gestures | 5 | 1 | 17 | 94% | **y** |
| including those behaviorally diagnosed as in a Vegetative State/Unresponsive Wakefulness syndrome | 5 | 0 | 17 | 76% | **y** |
| to follow command and/or communicate | 5 | 0 | 17 | 88% | **y** |

***Legend***: Covert Awareness (CA), Cognitive Motor Dissociation (CMD), Non-behavioral MCS (MCS*), functional Locked-In Syndrome (fLIS), Median (Mdn), Interquartile range (IQR), sample size (n), Consensus (yes/y, no/n)

**Supplementary Table 3.** Agreement rates for names such as CA, CMD, MCS*, fLIS as well as for covert related names, according to geographic location (EU/UK, USA, Others) and background (neuroscientist)

| EU/UK | *Mdn* | *IQR* | *n* | *% agree* | *Consensus* |
|---|---|---|---|---|---|
| **After taking the survey (Round 2)** | | | | | |
| Covert Awareness (CA) | 5 | 1 | 34 | 94% | y |
| Cognitive Motor Dissociation (CMD) | 4 | 2 | 34 | 74% | n |
| Non-behavioral MCS (MCS*) | 2 | 2 | 34 | 35% | n |
| functional Locked-In Syndrome (fLIS) | 2 | 1 | 34 | 6% | y |
| **Covert related names** | | | | | |
| Covert awareness | 4 | 1 | 34 | 88% | n |
| Covert consciousness | 4 | 3 | 34 | 65% | n |
| Covert cognition | 2 | 2 | 34 | 29% | n |
| **USA** | | | | | |
| **After taking the survey (Round 2)** | | | | | |
| Covert Awareness (CA) | 5 | 1 | 22 | 82% | y |
| Cognitive Motor Dissociation (CMD) | 4 | 2 | 22 | 73% | n |
| Non-behavioral MCS (MCS*) | 2 | 2 | 22 | 27% | n |
| functional Locked-In Syndrome (fLIS) | 2 | 2 | 20 | 20% | n |
| **Covert related names** | | | | | |
| Covert awareness | 4 | 1 | 22 | 77% | n |
| Covert consciousness | 5 | 2 | 22 | 73% | n |
| Covert cognition | 3 | 2 | 22 | 45% | n |
| **Others** | | | | | |
| **After taking the survey (Round 2)** | | | | | |
| Covert Awareness (CA) | 5 | 0 | 15 | 80% | y |
| Cognitive Motor Dissociation (CMD) | 4 | 1 | 15 | 73% | n |
| Non-behavioral MCS (MCS*) | 2 | 2 | 15 | 20% | n |
| functional Locked-In Syndrome (fLIS) | 2 | 2 | 15 | 13% | n |

| | | | | | |
|---|---|---|---|---|---|
| **Covert related names** | | | | | |
| Covert awareness | 5 | 1 | 15 | 80% | y |
| Covert consciousness | 4 | 2 | 15 | 60% | n |
| Covert cognition | 3 | 2 | 15 | 27% | n |
| **Neuroscientist** | | | | | |
| **After taking the survey (Round 2)** | | | | | |
| Covert Awareness (CA) | 5 | 1 | 17 | 82% | y |
| Cognitive Motor Dissociation (CMD) | 4 | 1 | 17 | 76% | n |
| Non-behavioral MCS (MCS*) | 2 | 3 | 17 | 47% | n |
| functional Locked-In Syndrome (fLIS) | 2 | 1 | 17 | 6% | y |
| **Covert related names** | | | | | |
| Covert awareness | 5 | 1 | 17 | 88% | y |
| Covert consciousness | 4 | 2 | 17 | 53% | n |
| Covert cognition | 2 | 2 | 17 | 29% | n |

***Legend***: Covert Awareness (CA), Cognitive Motor Dissociation (CMD), Non-behavioral MCS (MCS*), functional Locked-In Syndrome (fLIS), Median (Mdn), Interquartile range (IQR), sample size (n), Consensus (yes/y, no/n)
